# The spread of COVID-19 outbreak in the first 120 days: a comparison between Nigeria and seven other countries

**DOI:** 10.1186/s12889-020-10149-x

**Published:** 2021-01-12

**Authors:** Ayo Stephen Adebowale, Adeniyi F. Fagbamigbe, Joshua O. Akinyemi, Olalekan K. Obisesan, Emmanuel J. Awosanya, Rotimi F. Afolabi, Selim A. Alarape, Sunday O. Obabiyi

**Affiliations:** 1 Department of Epidemiology and Medical Statistics, Faculty of Public Health, College of Medicine, University of Ibadan, Ibadan, Nigeria; 2 Infectious Diseases Institute, College of Medicine, University of Ibadan, Ibadan, Nigeria; 3 Department of Statistics, Faculty of Science, University of Ibadan, Ibadan, Nigeria; 4 Department of Veterinary Public Health and Preventive Medicine, Faculty of Veterinary Medicine, University of Ibadan, Ibadan, Nigeria; 5 Population and Health Research Entity, Faculty of Humanities, North-West University, Mmabatho, South Africa; 6 Department of Mathematics, Faculty of Science, University of Ibadan, Ibadan, Nigeria

**Keywords:** COVID-19, Situation assessment, Model fit, Nigeria

## Abstract

**Background:**

COVID-19 is an emerging public health emergency of international concern. The trajectory of the global spread is worrisome, particularly in heavily populated countries such as Nigeria. The study objective was to assess and compare the pattern of COVID-19 spread in Nigeria and seven other countries during the first 120 days of the outbreak.

**Methods:**

Data was extracted from the World Bank’s website. A descriptive analysis was conducted as well as modelling of COVID-19 spread from day one through day 120 in Nigeria and seven other countries. Model fitting was conducted using linear, quadratic, cubic and exponential regression methods (α=0.05).

**Results:**

The COVID-19 spread pattern in Nigeria was similar to the patterns in Egypt, Ghana and Cameroun. The daily death distribution in Nigeria was similar to those of six out of the seven countries considered. There was an increasing trend in the daily COVID-19 confirmed cases in Nigeria. During the lockdown, the growth rate in Nigeria was 5.85 (R^2^=0.728, *p*< 0.001); however, it was 8.42 (R^2^=0.625, *p*< 0.001) after the lockdown was relaxed. The cubic polynomial model (CPM) provided the best fit for predicting COVID-19 cumulative cases across all the countries investigated and there was a clear deviation from the exponential growth model. Using the CPM, the predicted number of cases in Nigeria at 3-month (30 September 2020) was 155,467 (95% CI:151,111-159,824, *p*< 0.001), all things being equal.

**Conclusions:**

Improvement in COVID-19 control measures and strict compliance with the COVID-19 recommended protocols are essential. A contingency plan is needed to provide care for the active cases in case the predicted target is attained.

## Background

The novel Coronavirus disease (COVID-19) is one of the diseases that have constituted global threats in human history. The global burden of disease attributable to COVID-19 is enormous [1–3]. It is challenging to control the pandemic because of the high epidemic potential of the disease and the lack of potent vaccines to ramp up the herd immunity of populations. Thus, COVID-19 has spread to almost all countries of the world including Nigeria [3]. The first case was confirmed in Nigeria on 27 February 2020 [2] and by day 120 (25 June 2020), the cumulative cases had risen to 22,614 [2]. There was a suspicion that the reported confirmed cases did not reflect the true situation because of the low level of testing.

COVID-19 testing rate in Nigeria has been considerably low compared to other African countries and countries with similar population size. This has been attributed sometimes to delays in kits and reagent supplies during the border closures although, the Nigeria Centre for Disease Control (NCDC) claimed that the strict adherence to COVID-19 protocol and guidelines was partly responsible for the low level of testing [2]. However, the number of testing centres has now increased and the COVID-19 testing capacity has improved tremendously across the country.

Nigeria is a low-income country with a fragile health system. The control strategies instituted against COVID-19 spread in the country appeared insufficient as the number of daily cases continued to record new highs. With densely populated cities and towns, infectious diseases spread would be challenging to contain. In this study, we attempted to monitor and assess the COVID-19 situation in Nigeria using the 120-day data for confirmed cases. In addition, we compared the disease spread patterns, death, and the fitted model with seven other countries. Two research questions were consequently addressed: (i) How does COVID-19 spread and mortality compare with other countries in the first 120 days? (ii) Given the prevailing testing regime, interventional efforts and all other things being equal, what is the 3-month projected cumulative cases of COVID-19?

## Methods

The cross-sectional study was conducted in Nigeria using data from February 27 to June 25, 2020. Nigeria has a population of about 200 million, many international land borders and a few international airports. As at the time of writing this report, situation assessment showed that the compliance with the government directive on precautionary measures against COVID-19 was low in Nigeria [2].

The secondary data utilised for this study was extracted from the World Bank Database on COVID-19 [1]. Seven other countries (Ghana, Egypt, Cameroun, South Africa, Bangladesh, Indonesia and Mexico) were purposively selected from Africa, Asia and South America. These countries were selected because they experienced the outbreak of COVID-19 almost concurrently as Nigeria and the magnitude of the cumulative confirmed cases was relatively similar to that of Nigeria on the World COVID-19 table as at the time of data extraction for this study. Additionally, the countries were similar in terms of their economic development being classified as Low-Middle Income countries by the World Bank. The extracted data were restricted to the first 120 days of the outbreak of COVID-19 in these countries. The population figure, population density [4] and number of confirmed cases of COVID-19 as at day 120 of the outbreak in the selected countries are represented in Table 1.

**Table 1:** Distribution of the population figure, population density and COVID-19 confirmed cases by selected countries^a^

Country	Population Figure	Population Density (person/km^**2**^)	Total COVID-19 Confirmed Cases
Bangladesh	164,689,353	1116	149,258
Egypt	102,334,903	102.2	41,304
Cameroun	26,545,868	55.84	12,192
Nigeria	206,139,587	223.2	22,614
South Africa	59,308,689	48.65	159,333
Mexico	128,932,753	65.63	231,770
Ghana	31,072,970	130.26	18,134
Indonesia	273,523,620	143.14	54,010

Sources: United Nations, Department of Economic and Social Affairs, Population Division. World Population Prospects: The 2019 Revision; https://www.ecdc.europa.eu/en/publications-data

^a^As at day 120 of the outbreak in the respective countries

Data were presented with charts and line graphs. Four classes of regression model were used to fit the cumulative data on COVID-19. These are: linear, quadratic, cubic and exponential models. The equations representing each model are presented in Table 2.

**Table 2:** The four predictive models

Model	Equation	df
Linear	*N*_*x*_ = *a* + *bx* + *ε*	1
Quadratic	*N*_*x*_ = *c* + *dx* + *ex*^2^ + *ε*	2
Cube polynomial	*N*_*x*_ = *f* + *gx* + *hx*^2^ + *jx*^3^ + *ε*	3
Exponential	*N*_*x*_ = *kexp*(*mx*) + *ε*	1

where a, c, f and k are intercepts; b, d, e, g, h, j and m are slopes which are estimated by least square estimation, *x* is the time-point and ɛ’s are mutually uncorrelated random errors with mean (0) and common variance (*σ*^2^); *df* is the degree of freedom

Further, the model with the best fit was used to predict a 3-month (30 September 2020) cumulative confirmed COVID-19 cases in Nigeria.

## Results

**Fig. 1:**
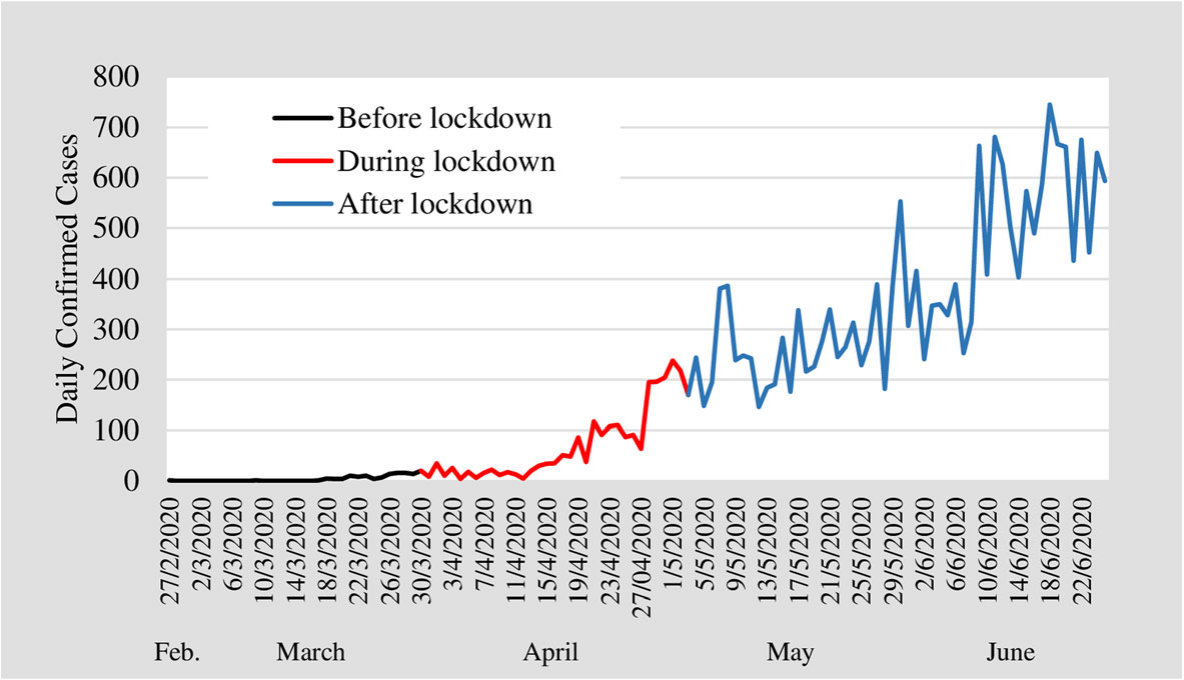
Daily COVID-19 confirmed cases as at the first 120 days of the outbreak in Nigeria. A graphical display of the daily confirmed cases of COVID-19 in Nigeria

The data as presented in Fig. 1 shows an increasing trend in the daily confirmed cases of COVID-19 in Nigeria, from day 1 through day 120. There was a consistent increase in the number of cases after the relaxation of lockdown measures. During the lockdown, the growth rate of COVID-19 in Nigeria was 5.85 (95% CI:5.41,6.29; *R*^2^=0.728; *p*< 0.001) and this increased to 8.42 (95% CI:8.41,8.43; *R*^2^=0.625; *p*< 0.001) after the lockdown was relaxed.

**Fig. 2:**
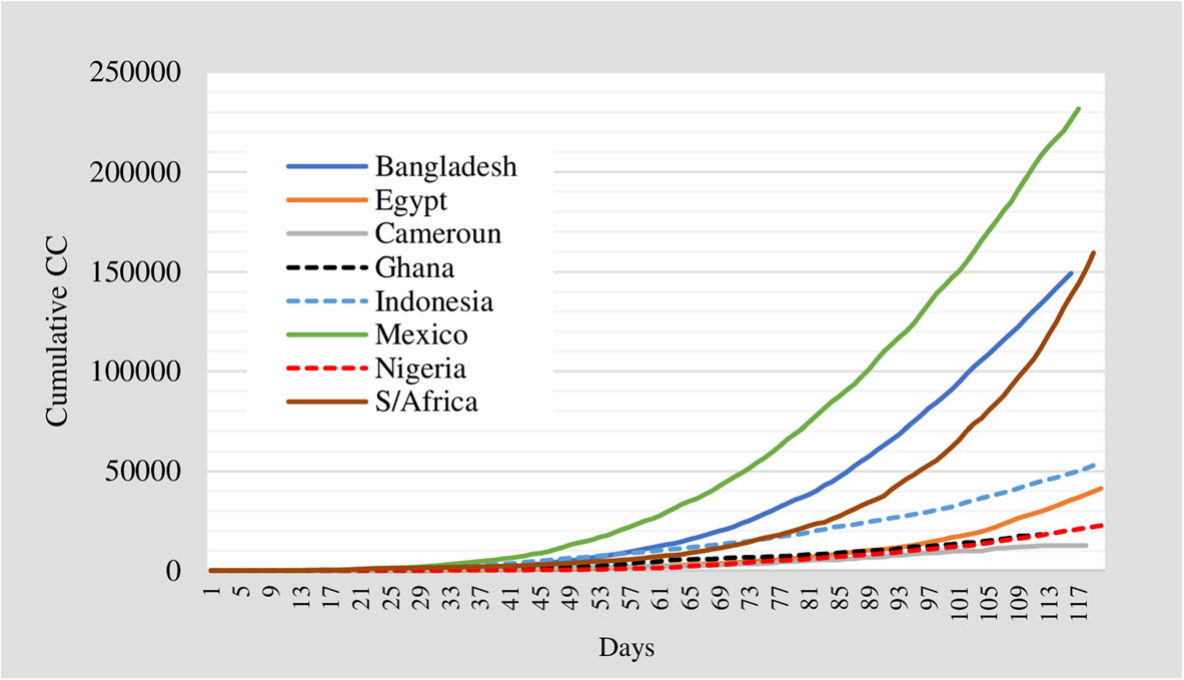
COVID-19 cumulative confirmed cases in selected countries as at the first 120-day of the outbreak. A graphical presentation of the cumulative daily confirmed cases of COVID-19 in the first 120 days after the outbreak in eight countries

Figure 2 shows the cumulative daily confirmed cases of COVID-19 in the first 120 days after the outbreak in eight countries. The pattern of Nigeria’s cumulative daily confirmed cases aligned with three (Ghana, Egypt and Cameroun) of the seven countries, particularly within the first 97 days. The trajectory of the spread of COVID-19 in Nigeria aligned perfectly with Ghana’s pattern and slightly differs from the pattern exhibited by Cameroun after the 97th day. There was a clear difference in the pattern observed between Nigeria and Mexico, Bangladesh, South Africa and Indonesia respectively.

**Fig. 3:**
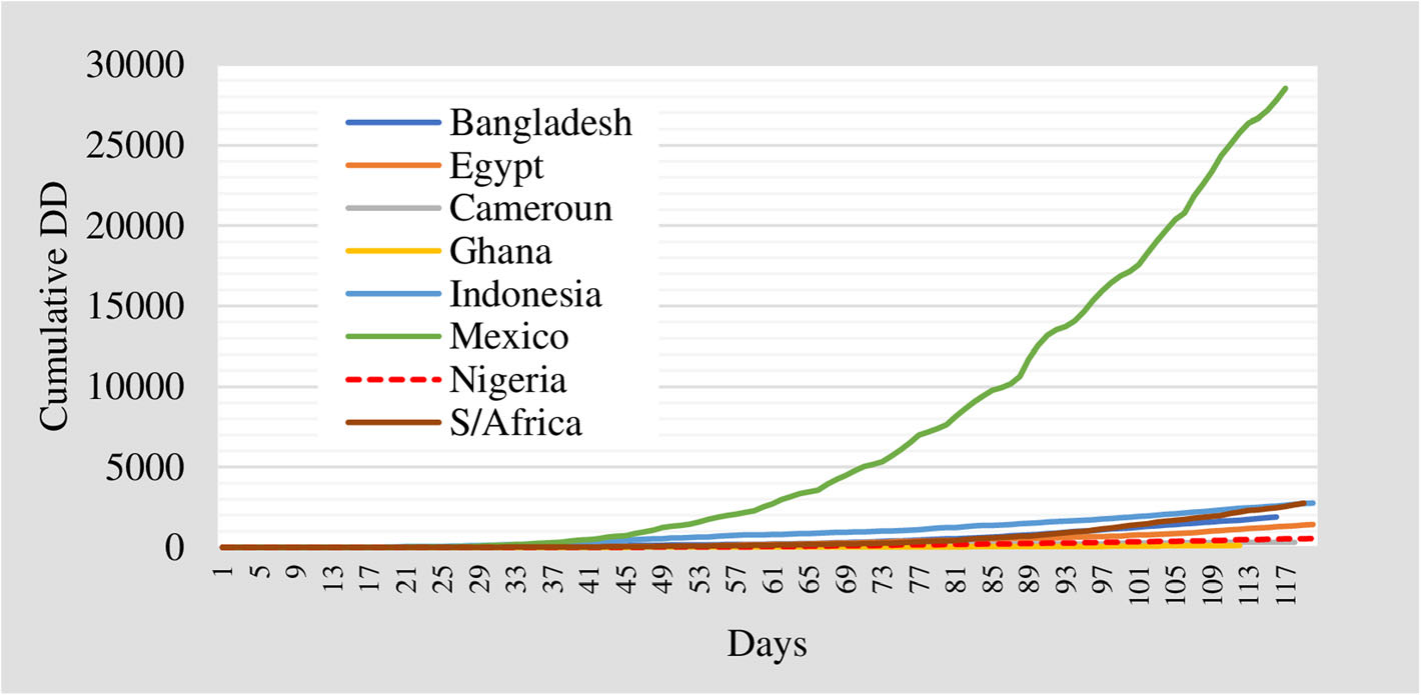
COVID-19 cumulative daily deaths for the first 120 days in selected countries. A figure showing the distribution of COVID-19-related deaths within the first 120 days after the outbreak

The distribution of deaths associated with COVID-19 within the first 120 days after the outbreak shows that the observed pattern for Nigeria was nearly the same with six of the seven countries compared. In particular, the Nigeria’s pattern was identical to other countries in Central and West Africa (Cameroun and Ghana) (Fig. 3).

The summary of the model and estimated parameters are shown in Table 3. Across all the eight countries included in the analyses, the Cubic Polynomial Model (CPM) was identified as the best fit to model COVID-19 data. The CPM perfectly fits the Mexico data with an R-square of 100%. The R-square for Nigeria was 99.9%, an indication that 99.9% of the variation in the cumulative daily confirmed cases of COVID-19 in Nigeria can be explained by the model with time as the main covariate. The predictive models for Nigeria are: Linear: *N*_*x*_ =  − 4937.222 + 164.210*x*, Quadratic: *N*_*x*_ = 1748.194 − 164.581*x* + 2.717*x*^2^, Cubic: *N*_*x*_ = 151.233 − 9.415*x* − 0.475*x*^2^ + 0.018*x*^3^, Exponential: *N*_*x*_ = 2.245 *exp* (0.090*x*) (Table 3).

**Table 3:** Model summary and parameter estimates

Country	Summary	Parameter Estimates			
	***R Square***	***Constant***	***β***_***1***_	***β***_***2***_	***β***_***3***_
**Bangladesh**					
Linear	0.767^a^	−33,018.645	1122.635		
Quadratic	0.987^a^	13,105.539	− 1222.663	20.045	
Cubic	0.999^a^	− 313.915	124.858	−8.625	0.163
Exponential	0.915^a^	9.628	0.098		
**Egypt**					
Linear	0.699^a^	− 8039.316	256.061		
Quadratic	0.956^a^	4274.644	− 349.544	5.005	
Cubic	0.995^a^	− 1566.637	218.015	−6.673	0.064
Exponential	0.893^a^	2.352	0.094		
**Cameroun**					
Linear	0.860^a^	− 2845.963	112.376		
Quadratic	0.992^a^	591.439	−59.494	1.444	
Cubic	0.993^a^	146.269	−15.528	.525	0.005
Exponential	0.820^a^	15.465	0.069		
**Ghana**					
Linear	0.894^a^	− 3633.083	156.014		
Quadratic	0.995^a^	268.922	−49.354	1.817	
Cubic	0.995^a^	183.709	− 40.501	1.622	0.001
Exponential	0.853^a^	29.047	0.068		
**Indonesia**					
Linear	0.887^a^	−10,357.170	422.333		
Quadratic	0.997^a^	1439.768	− 157.844	4.795	
Cubic	0.999^a^	− 550.761	35.562	.815	0.022
Exponential	0.782^a^	56.394	0.069		
**Mexico**					
Linear	0.823^a^	−50,669.386	1832.606		
Quadratic	0.995^a^	14,390.013	− 1447.700	27.799	
Cubic	1.000^a^	1958.116	− 209.705	1.682	0.148
Exponential	0.868^a^	97.206	0.079		
**Nigeria**					
Linear	0.785^a^	−4937.222	164.210		
Quadratic	0.991^a^	1748.194	−164.581	2.717	
Cubic	0.999^a^	151.233	−9.415	−.475	0.018
Exponential	0.911^a^	2.245	0.090		
**South Africa**					
Linear	0.667^a^	−29,085.662	929.637		
Quadratic	0.941^a^	17,807.574	− 1395.647	19.377	
Cubic	0.995^a^	− 7413.468	1074.917	−31.878	0.285
Exponential	0.865^a^	50.331	0.075		

^a^Significant at 0.1%

**Fig. 4:**
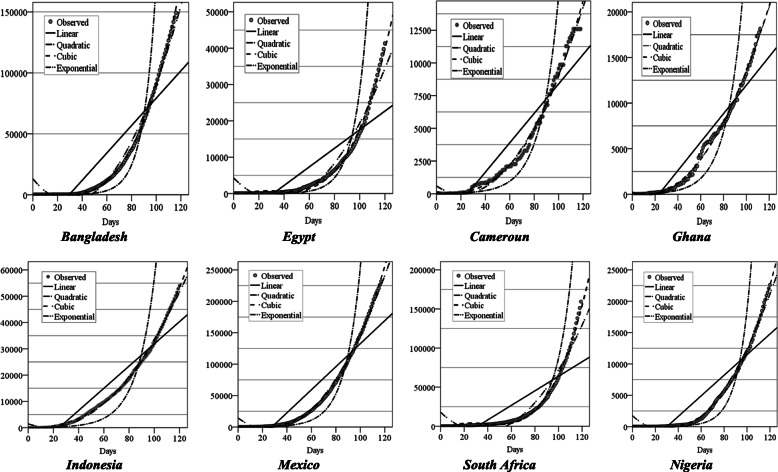
Predictive model of cumulative cases of COVID-19 in Nigeria. A graphical display of the observed and predicted cumulative cases of COVID-19 in the selected countries, using the four models

The data as presented in Fig. 4 depict the observed and predicted cumulative cases of COVID-19 in the selected countries as at day 120 of the outbreak. The trajectory of observed COVID-19 cumulative cases in Nigeria deviated from the exponential and linear models but perfectly fits the quadratic and cubic regression models. The exponential model fits the data for South Africa and, to some extent, Egypt in the first 70 days of the outbreak in these countries. The simple linear regression model did not fit the data for any of the studied countries.

**Fig. 5:**
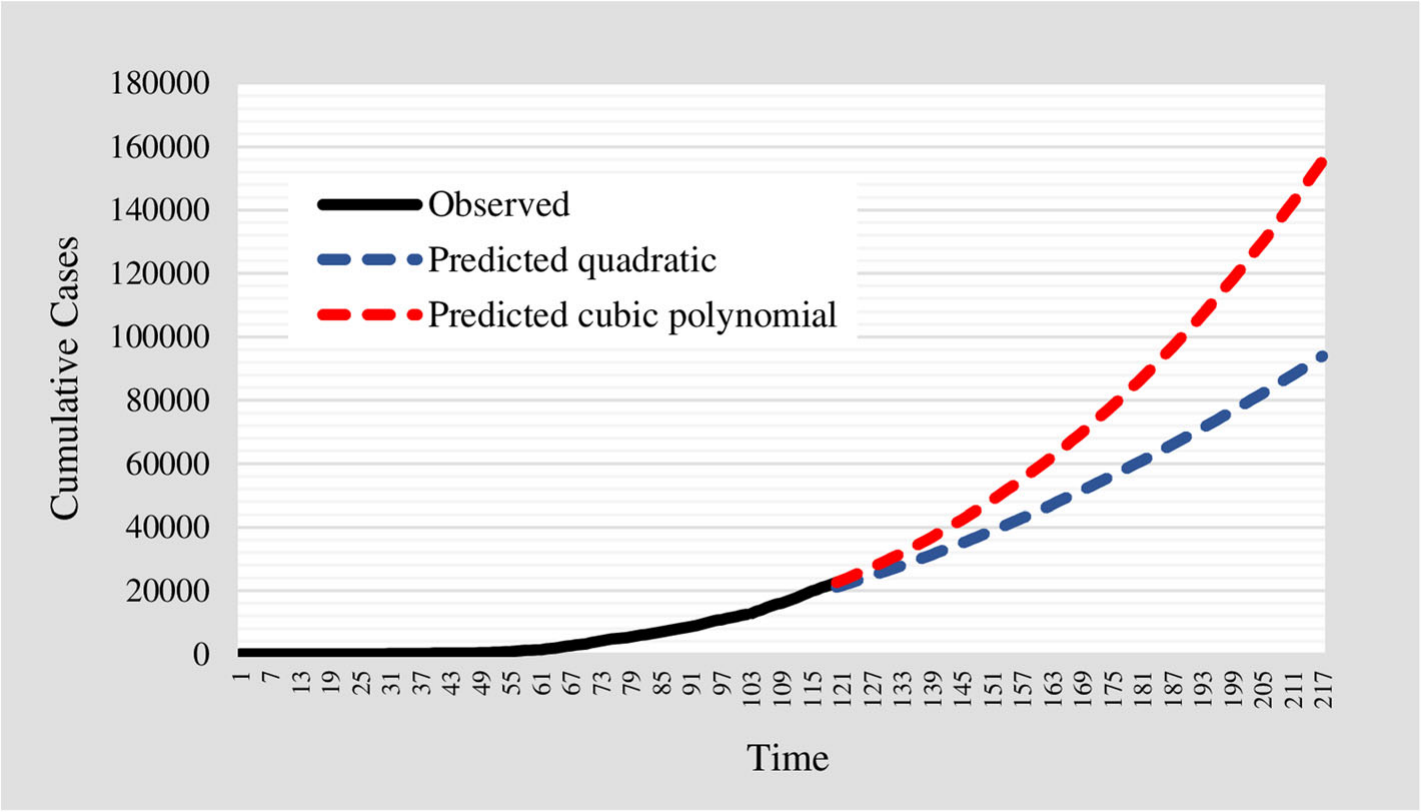
Observed and projected COVID-19 cumulative cases in Nigeria. A graphical display of the observed and estimated values of cumulative cases of COVID-19 in Nigeria using both quadratic and cubic polynomial models

The observed and estimated values of cumulative cases of COVID-19 in Nigeria using both quadratic and cubic polynomial models are presented in Fig. 5. The 30 September 2020 predicted COVID-19 cumulative case was: 93,988 (95% CI: 91,209-96,767) – Quadratic; 155,467 (95% CI:151,111-159,824) – Cubic.

## Discussion

The goal of this paper was to assess the COVID-19 spread and attendant deaths in Nigeria and selected countries during the first 120 days of outbreaks. First, our analysis showed the spread of the pandemic increased following the relaxation of lockdowns. Both spread and mortality patterns in Nigeria compared closely with other African countries (Ghana, Cameroun and Egypt). Lastly, the findings suggested that different predictive models fitted the data for different countries.

In this study, the COVID-19 data in Nigeria showed an increasing trend. The epidemic curve for Nigeria differed from the typical propagated epidemic curve that would have been expected for COVID-19 and other infectious diseases with person-to-person mode of transmission. COVID-19 reproduction number appears to gain traction with time as estimated to be from 1.4 to 2.5, 3.6 to 4.0, and 2.24 to 3.58 from earlier studies [3, 5–7]. This indicates that the infection rate continues to increase. Therefore, the observed pattern found in our study agrees with the known pattern of spread of the disease. We also found that a higher number of cases was reported after the relaxation of lockdown than was reported during the lockdown period. The increased spread of the disease after the relaxation of lockdown could be due to poor adherence to the recommended preventive measures in Nigeria. This may be expected because when restrictive measures are lifted, exposures to disease risks become higher. Consequently, an increased number of infections is likely to follow. This is in tandem with evidence from developed countries where the transmission dynamics and effectiveness of control measures have been rigorously studied [8, 9]. However, another plausible explanation for the increase in the number of confirmed cases could be the improved testing capacity in the country which coincided with the lockdown relaxation. Compared with Ghana and South Africa, the testing capacity in Nigeria was generally low. While Nigeria had only 2755 persons tested for COVID-19 per 1,000,000 people, the estimate was 16,206 and 76,067 for Ghana and South Africa respectively [10].

We found a similar pattern in the number of cumulative cases of COVID-19 in Nigeria, Ghana and Cameroon possibly due to similarity in the capacity for testing in the first 120 days of the disease outbreak. Conversely, a difference was observed in the pattern exhibited by Nigeria compared to four of the seven countries investigated (Mexico, Bangladesh, South Africa and Indonesia). As at day 120 in these four countries, the total COVID-19 tests per 1 million population was strikingly higher than that of Nigeria over the same period [1, 10]. Only Indonesia had a population size that was similar to that of Nigeria while the other countries had a considerably lower population than that of Nigeria. Mexico, Bangladesh, South Africa and Indonesia had environmental factors such as temperature and humidity that were comparable to that of Nigeria [11, 12]. Thus, the reasons that could explain the differences in disease spread pattern between these countries and Nigeria is not immediately clear except for the differences in COVID-19 testing rate. The implication is that community testing has not commenced fully in Nigeria as it is done in South Africa. The disease currently exhibited a sporadic cluster of local transmission in Nigeria.

We further found that the distribution of COVID-19-associated deaths observed for Nigeria was comparable to six of the seven countries investigated and aligned perfectly with the Cameroun and Ghana patterns. COVID-19-related death trajectory in Mexico may be explained by the higher number of observed COVID-19 cases recorded within the study period compared to other countries. The similarity in COVID-19 deaths between Nigeria, Ghana and Cameroon may be attributed to other factors different from the COVID-19 testing capacity and case management capabilities.

Cubic Polynomial Model (CPM) was identified as the best fit model among the four models used in this study. Next to the CPM is the quadratic model (QM). The CPM and QM have been identified in some previous studies as the best predictive models for some infectious diseases including Ebola and COVID-19 [13, 14]. None of the country data was suited for the exponential model except South Africa, which was suitable for the first 70 days of the outbreak. A similar observation was reported previously on the suitability of the exponential model for fitting the epidemic curve of infectious diseases [15]. Nonetheless, differences in the levels and modes of testing across countries could be responsible for South Africa’s exemption. In South Africa, a community testing approach was instituted early unlike in other African countries such as Nigeria, Ghana and Cameroon. In addition, the marked differences in atmospheric and environmental conditions between countries may constitute potential explanatory variables [11, 12].

In our study, the predicted COVID-19 cumulative case for 30 September 2020 using QM and CPM was 93,988 and 155,467 respectively. This is premised on the assumption that the present COVID-19 testing capacity and the level of compliance with the preventive measures to mitigate the spread is sustained. The wide gap between the two estimates could be linked to differences in the equations governing the use of QM and CPM, which implied that different parameters were used for the estimation. In this study, the model with the best fit differed across countries indicating variations in the epidemiological contexts, transmission dynamics and control efforts. Although some of these countries shared similarities in demographic and developmental profile, there were potential differences in other factors such as testing capacity, risk profile, enforcement of containment measures and the level of exposure to infected individuals.

The public health implication of our study is that there is a need for adequate emergency preparedness. The identified trajectory of COVID-19 infection in Nigeria is an impetus for increased surveillance, enhanced testing capacity and proactive planning for clinical management of cases as well as psychosocial management of discharged cases. Also, the preventive measures may have to be strengthened for containment of disease spread in Nigeria and the other countries.

### Limitations

The Nigeria data was premised on testing suspected cases who reported at the testing centres or symptomatic individuals who called the NCDC response team lines at their various homes for help. The differences in the scale of testing and the environmental conditions in different countries should be considered in interpreting our findings. This is because evidence suggests a relationship between weather conditions and transmission risks of COVID-19 [11, 12]. Inaccessibility to data on socio-demographic profile and health history of the COVID-19 patients and survivors limited the opportunity to perform further statistical and mathematical modelling.

## Conclusions

The spread of COVID-19 is increasing daily and the projection provides insight into what the situation could be in days ahead in Nigeria. Thus, enhanced emergency preparedness and contingency plans to mitigate the COVID-19 spread are urgently required. There is a need to increase testing capacities both at State and Local government levels as current testing is limited. Improvement in COVID-19 control measures and strict compliance with the COVID-19 recommended protocols are strongly recommended. Preparation should be made for the case management of COVID-19 cases in Nigeria.

## Data Availability

Data sharing does not apply to this article as no new data were created or analysed in this study. The data is publicly available and openly accessible at https://www.ecdc.europa.eu/en/publications-data [1].

## Abbreviations

SARS-CoV-2: Severe acute respiratory syndrome coronavirus 2
WHO: World health organization
NCDC: Nigeria centre for disease and control
ECDC: European centre for disease prevention and control
R^2^: Coefficient of determination
FCT: Federal capital territory
CPM: Cubic polynomial model

## References

[1] European Centre for Disease Prevention and Control (2020). Publications & data.

[2] Nigeria Centre for Disease Control. 2020. https://ncdc.gov.ng/diseases/sitreps/?cat=14&name=An%20update%20of%20COVID-19%20outbreak%20in%20Nigeria. Accessed 2 July 2020.

[3] World Health Organisation (2020). Coronavirus (COVID-19) events as they happen.

[4] United Nations, Department of Economic and Social Affairs, Population Dynamics. World population prospects: the 2019 revision: United Nations; 2020. https://population.un.org/wpp/. Accessed 11 July 2020.

[5] Read JM, Bridgen JRE, Cummings DAT, Ho A, Jewell CP. Novel coronavirus 2019-nCoV: early estimation of epidemiological parameters and epidemic predictions. doi: 10.1101/2020.01.23.20018549. 10.1098/rstb.2020.0265 PMC8165596 34053269

[6] Majumder M, Mandl KD. Early transmissibility assessment of a novel coronavirus in Wuhan, China. SSRN Electron J. 2020. 10.2139/ssrn.3524675.

[7] Imai N, Cori A, Dorigatti I, Baguelin M, Donnelly CA, Riley S (2020). Report 3: transmissibility of 2019-nCoV.

[8] Fang Y, Nie Y, Penny M (2020). Transmission dynamics of the COVID-19 outbreak and effectiveness of government interventions: a data-driven analysis. J Med Virol.

[9] Liu P, He S, Rong L, Tang S (2020). The effect of control measures on COVID-19 transmission in Italy: comparison with Guangdong province in China. Infect Dis Poverty.

[10] Wordometer COVID-19 coronavirus pandemic: reported cases and deaths by country, territory, or conveyance. https://www.worldometers.info/coronavirus/#countries. Accessed 11 June 2020.

[11] Ogaugwu C, Mogaji H, Ogaugwu E, Nebo U, Okoh H, Agbo S, et al. Effect of weather on COVID-19 transmission and mortality in Lagos, Nigeria. Scientifica (Cairo). 2020;2562641. 10.1155/2020/2562641. 10.1155/2020/2562641 PMC7443043 32855836

[12] Prata DN, Rodrigues W, Bermejo PH (2020). Temperature significantly changes COVID-19 transmission in (sub)tropical cities of Brazil. Sci Total Environ.

[13] Valeri L, Patterson-Lomba O, Gurmu Y, Ablorh A, Bobb J, Townes FW (2016). Predicting subnational ebola virus disease epidemic dynamics from sociodemographic indicators. PLoS One.

[14] Malato G (2020). Covid-19 infection in Italy. Mathematical models and predictions.

[15] Solis FJ, Tapia B, Romero JV, Moreno J (2005). Quadratic infectious diseases mathematical models: chronic states, sanity levels, and treatment. Math Comput Model.

